# Fine-mapping sequence mutations with a major effect on oligosaccharide content in bovine milk

**DOI:** 10.1038/s41598-019-38488-9

**Published:** 2019-02-14

**Authors:** Zhiqian Liu, Tingting Wang, Jennie E. Pryce, Iona M. MacLeod, Ben J. Hayes, Amanda J. Chamberlain, Christy Vander Jagt, Coralie M. Reich, Brett A. Mason, Simone Rochfort, Benjamin G. Cocks

**Affiliations:** 1 Agriculture Victoria Research, AgriBio, 5 Ring Road, Bundoora, Victoria 3083 Australia; 2 School of Applied Systems Biology, La Trobe University, Bundoora, Victoria 3083 Australia; 3 Queensland Alliance for Agriculture and Food Innovation, Centre for Animal Science, University of Queensland, Queensland, Australia

## Abstract

Human milk contains abundant oligosaccharides (OS) which are believed to have strong health benefits for neonates. OS are a minor component of bovine milk and little is known about how the production of OS is regulated in the bovine mammary gland. We have measured the abundance of 12 major OS in milk of 360 cows, which had high density SNP marker genotypes. Most of the OS were found to be highly heritable (h^2^ between 50 and 84%). A genome-wide association study allowed us to fine-map several QTL and identify candidate genes with major effects on five OS. Among them, a putative causal mutation close to the ABO gene on Chromosome 11 accounted for approximately 80% of genetic variance for two OS, *N*-acetylgalactosaminyllactose and lacto-*N*-neotetraose. This mutation lies very close to a variant associated with the expression levels of ABO. A third QTL mapped close to ST3GAL6 on Chromosome 1 explaining 33% of genetic variation of an abundant OS, 3′-sialyllactose. The presence of major gene effects suggests that targeted marker-assisted selection would lead to a significant increase in the level of these OS in milk. This is the first attempt to map candidate genes and causal mutations for bovine milk OS.

## Introduction

Oligosaccharides (OS) are a class of carbohydrates containing 3–15 monomer units. The most frequent monomers are glucose, fructose, galactose, and sialic acid. The role of OS in promoting human health is widely known. Acting as prebiotics, OS stimulate the growth of beneficial bifidobacteria in the colon^1,2^. OS can also prevent infection by inhibiting the adhesion of pathogens to the intestinal mucosal surface^3^. Furthermore, sialic acid, a component of milk OS, is essential for brain development and cognitive function of neonates^4^. Indeed, OS are one of the major components of human milk^5^.

Bovine milk is a staple drink and is also the most common ingredient in infant formulas. Bovine milk OS composition and content has been the subject of numerous studies in the past decade. Over 40 OS have been identified thus far in bovine milk^6–8^, but their overall concentrations are much lower compared to human milk OS^5^. As a result, fructooligosaccharides (FOS) extracted from plants and galactooligosaccharides (GOS) which are enzymatically synthesised are frequently added to infant formulas to mimic the functions of human milk OS^9,10^.

Bovine milk OS are structurally much closer to human milk OS than FOS and GOS^2^, so bovine milk OS would be a better replacement than FOS and GOS to human milk OS if their concentrations could be substantially increased. In addition, as bovine milk is consumed by the majority of the population in many western countries, increasing its OS content could also increase the uptake of OS by a large number of people worldwide. Many of the health benefits that milk OS provide for infants are expected to be equally applicable to humans of all ages^2^.

Bovine milk OS concentration has been investigated in relation to cow breed, animal diets and stage of lactation. Sundekilde *et al*.^7^ reported that Jersey milk contained higher levels of sialylated and also complex neutral fucosylated OS, while Holstein milk contained higher levels of the less complex neutral OS. However, the overall inter-breed difference in OS content detected in this study was rather modest. Information on OS content in relation to animal diets is scarce and very limited data found in the literature appear to suggest that in contrast with milk fat and protein content, milk OS level is not influenced by animal diets^11^. So increasing milk OS through diet manipulation is unlikely to be a feasible option. The most extensively investigated factor in relation to milk OS content is probably the stage of lactation. A large number of studies conducted in different countries showed that OS content is much higher in colostrum and declines gradually with the progression of lactation^12–15^.

Significant cow to cow variation in colostrum and milk OS content has been observed in a few studies^7,11,15^. External factors, such as diets and stage of lactation, were the same for all cows in these experiments, therefore genetic variation was proposed as a possible cause for the difference in OS production across individual cows^7,11^. However, information on the inheritance of this trait is still lacking. To our knowledge, there have been no studies on genetic inheritance and association mapping for OS accumulation in bovine milk, although quantitative trait loci (QTLs) with major effects on milk yield, fat content, protein content and lactose content in dairy cattle have been widely reported^16–21^.

In this study, our first aim was to determine the genetic factors controlling OS abundance. The second aim was to detect QTLs affecting the abundance of major milk OS in 360 Holstein cows using a genome-wide association study (GWAS) and then to fine-map putative causal variants with imputed sequence data. We report here candidate causal variants, genomic regions and candidate genes that show significant association with the production of some of the major OS.

## Results

### Phenotypic correlation between different OS species

A total of 12 major OS present in mature milk was surveyed in this study; their names, composition and accurate masses are summarized in Table 1, whereas their detailed structures can be found in Lee *et al*.^22^. A pairwise correlation analysis was conducted using the raw dataset that contains the relative abundance of the 12 major OS for 360 cows. Several strong correlations in relative abundance (*r* > 0.6) were found across these OS (Table S-1, bold, Supporting Information). These correlated OS species may share the same biosynthesis pathway or have a direct precursor-product relationship.

**Table 1:** Major OS species investigated in this study.

Name (code)	Composition^a^	*m/z* ^b^
Triose	3 Hex	503.1612
3′-sialyllactose (3′-SL)	2 Hex, 1 NeuAc	632.2038
6′-sialyllactose (6′-SL)	2 Hex, 1 NeuAc	632.2038
6′-sialyl-N-acetyllactosamine (6′-SLN)	1 Hex, 1 HexNAc, 1 NeuAc	673.2304
Disialyllactose (DSL)	2 Hex, 2 NeuAc	923.2992
*N*-acetylgalactosaminyllactose (GNL)	2 Hex, 1 HexNAc	544.1878
3′-sialylgalactosyllactose (OS-A)	3 Hex, 1 NeuAc	794.2566
Lacto-*N*-pentaose (OS-B)	4 Hex, 1 HexNAc	868.2934
Lacto-*N*-neotetraose (OS-C)	3 Hex, 1 HexNAc	706.2406
Di-*N*-acetylhexosaminyltriose (OS-D)	3 Hex, 2 HexNAc	909.3200
3′-glycolylneuraminyllactose (OS-E)	2 Hex, 1 NeuGc	648.1987
3′-sialyl-*N*-acetylglucosaminyllactose (OS-F)	2 Hex, 1 HexNAc, 1 NeuAc	835.2832

^a^Hex: glucose or galactose; HexNAc: *N*-acetylglucosamine or *N*-acetylgalactosamine; NeuAc: *N*-acetylneuraminic acid (sialic acid); NeuGc: *N*-glycolylneuraminic acid.

^b^Calculated *m/z* values for deprotonated molecular ions (detected in negative ion mode).

### Genetic basis of OS traits

We first investigated the heritability of the OS traits to determine the proportion of the observed trait variation that is due to genetic factors rather than environmental variation. Table 2 shows that most of the OS traits are highly heritable, with estimates ranging between 50% and 84%, so the differences between cows are largely due to genetic factors.

**Table 2:** Heritability, genetic and phenotypic variance for 12 bovine milk OS.

OS	Genetic variance	Phenotypic variance	Heritability (s.e.)
Triose	1.82E + 11	2.97E + 11	0.61 (0.14)
3′-SL	2.4E + 13	4.67E + 13	0.51 (0.15)
6′-SL	4.05E + 12	6.07E + 12	0.67 (0.14)
GNL	6.63E + 11	9.82E + 11	0.67 (0.14)
6′-SLN	5.74E + 10	8.36E + 10	0.69 (0.15)
DSL	4.79E + 11	6.93E + 11	0.69 (0.14)
OS-A	1.15E + 12	1.7E + 12	0.68 (0.14)
OS-B	1.94E + 10	2.31E + 10	0.84 (0.14)
OS-C	3.34E + 10	6.06E + 10	0.55 (0.15)
OS-D	9.69E + 08	1.26E + 09	0.77 (0.32)
0S-E	5.53E + 10	1.41E + 11	0.39 (0.15)
OS-F	2.97E + 09	3.58E + 09	0.83 (0.14)

### Power of association studies to detect QTLs

#### False discovery rates (FDR)

To assess the performance of GWAS, we first calculated the FDR for all traits at four different *p*-value thresholds: *p* < 0.000001, *p* < 0.00001, *p* < 0.0001, and *p* < 0.001 (Table 3). For traits Triose, 6′-SL, 6′-SLN, DSL, OS-D, OS-E, and OS-F, the FDR rates are relatively high and at *p* < 0.000001 there were no SNP, or only one SNP detected, indicating that the GWAS for these traits lacks sufficient power. By contrast, the FDR for five other traits 3′-SL, GNL, OS-A, OS-B and OS-C are relatively low. Even under the threshold *p* < 0.000001, the FDR for GNL and OS-C are 0.2 and 0.3%, providing strong evidence that the GWAS for these traits has ample power to detect real QTL. We therefore report details of QTL discovery and fine-mapping with sequence variants for only these five traits with low FDR.

**Table 3:** Number of significant SNPs and FDR for 12 OS under four GWAS thresholds (*p* < 0.000001, *p* < 0.00001, *p* < 0.0001 and *p* < 0.001).

OS	Number of significant SNPs				FDR (%)			
	<0.000001	<0.00001	<0.0001	<0.001	<0.000001	<0.00001	<0.0001	<0.001
Triose	0	21	75	505	—	27	75	>100
3′-SL	6	12	127	591	9	47	45	96
6′-SL	0	0	46	583	—	—	>100	97
GNL	235	325	436	859	0.2	2	13	66
6′-SLN	0	0	36	357	—	—	>100	>100
DSL	0	0	58	493	—	—	97	>100
OS-A	4	13	56	410	14	43	>100	>100
OS-B	12	30	78	585	5	19	72	97
OS-C	171	274	435	880	0.3	2	13	64
OS-D	0	4	22	334	—	>100	>100	>100
OS-E	1	5	50	477	57	>100	>100	>100
OS-F	0	11	54	556	—	51	>100	>100

#### QQ plot

**Figure 1:**
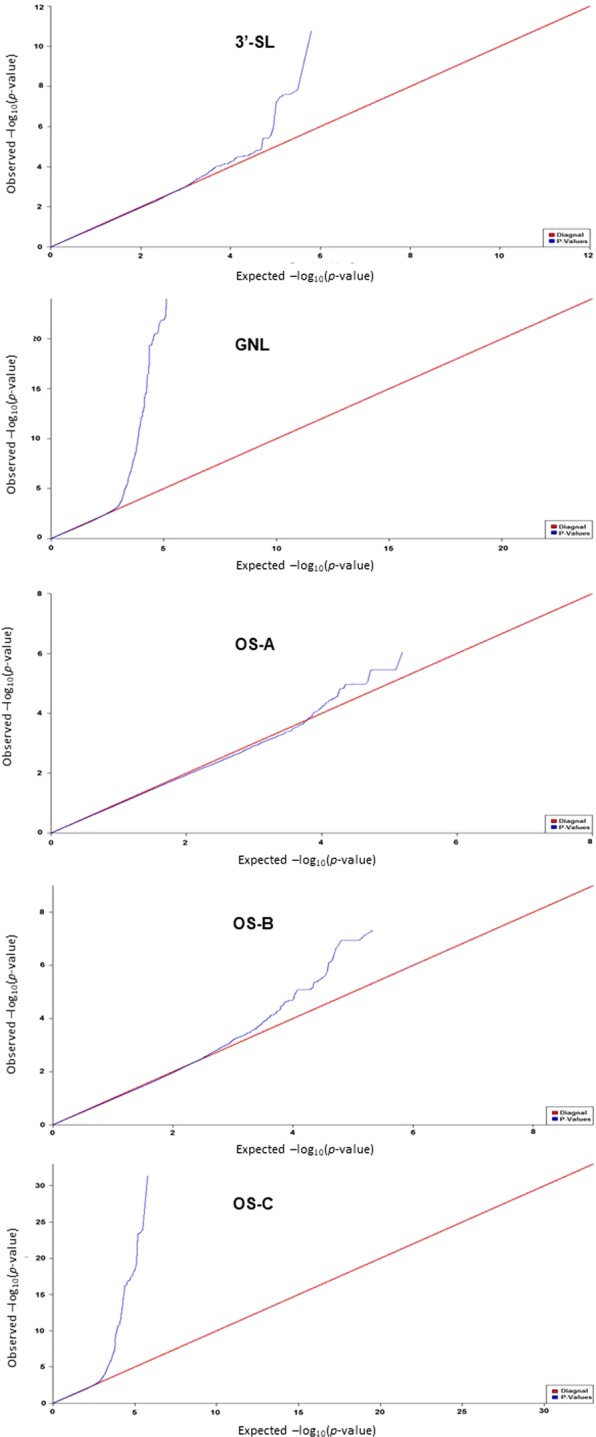
Quantile-quantile (QQ) plots of GWAS results for 3′-SL, GNL, OS-A, OS-B, and OS-C. Blue curve: observed −log_10_ (*p*-value); red line: expected −log_10_ (*p*-value) for null hypothesis.

Quantile-quantile (QQ) plots were used to further verify the quality of the above GWAS results for the five selected traits (3′-SL, GNL, OS-A, OS-B and OS-C). Figure 1 illustrates that the highest observed −log_10_ (*p*-values) for each of the five traits are higher than expected under the null hypothesis of no true association. In the case of GNL and OS-C, the observed −log_10_ (*p*-values) deviate considerably from the line corresponding to the null hypothesis, implying many SNPs are significantly associated with these two OS species.

### QTL discovery from HD SNP GWAS

**Figure 2:**
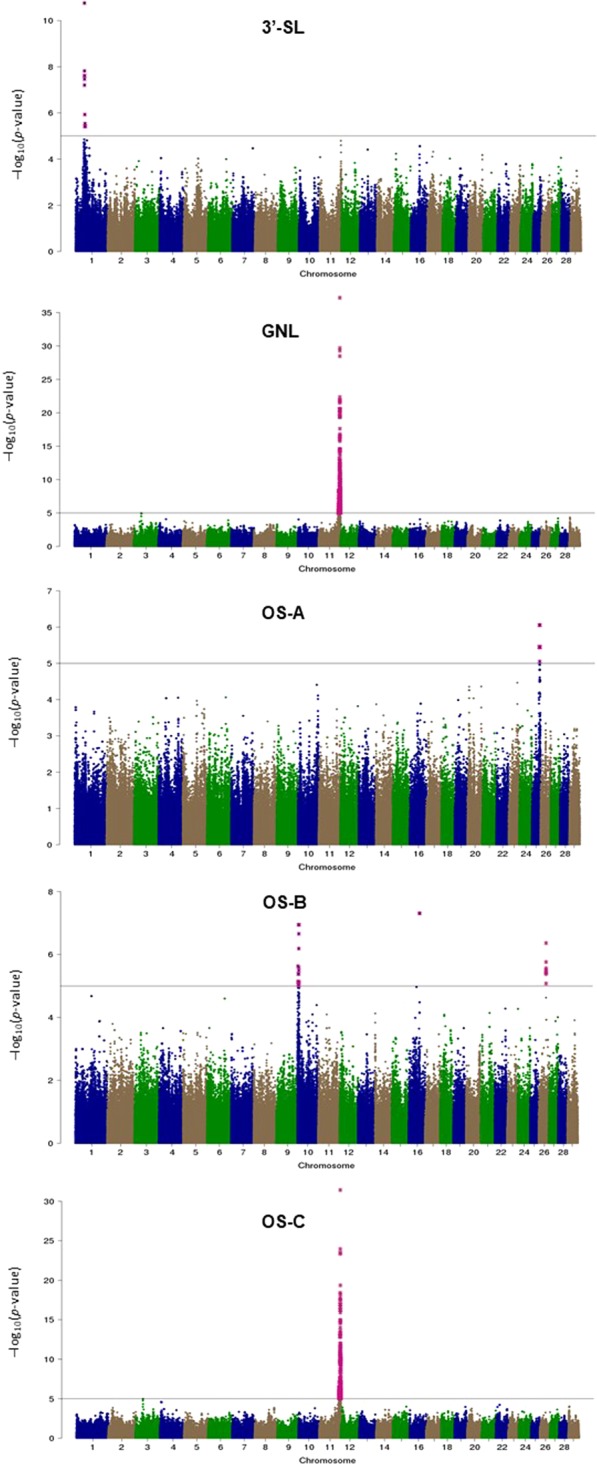
Manhattan plots of −log_10_ (*p*-value) for 3′-SL, GNL, OS-A, OS-B, and OS-C. The pink stars indicate strong QTL signals (*p* < 0.00001).

The GWAS results using HD SNP genotypes suggest the presence of several major QTL regions for the five OS traits with the lowest FDR (Fig. 2). Notably, a region on Chromosome 11 has a strong QTL signal affecting two correlated OS species GNL and OS-C (Fig. 2). There are some very sharp QTL peaks, which are likely to be close to the causal mutations. However, the most significant SNP are unlikely to be the causal mutations because they are SNP from the standard HD array, which is why we then carried out a GWAS with imputed sequence data for each of the chromosomes with the most significant SNP.

### Fine-mapping with sequence variants

For QTL regions shown in Fig. 2 with the lowest FDR, we undertook further fine-mapping association studies using imputed sequence variants on the relevant chromosomes. In theory, the causal mutations should be present in this sequence data, but it is difficult to pinpoint causal mutations in a GWAS because there are often strong associations between neighbouring alleles (linkage disequilibrium - LD). We therefore investigated LD between the most significant SNP and the remaining SNP in the region to more clearly define the extent of regions with strong LD and thus to identify putative candidate genes and putative causal mutations across these regions of strong LD. The LD statistic (*r*^2^) provides a basis for more precisely defining the most likely region for the causal mutation. The LD *r*^2^ was estimated by the squared correlation between pairwise genotype allele counts using PLINK software^23^.

**Figure 3:**
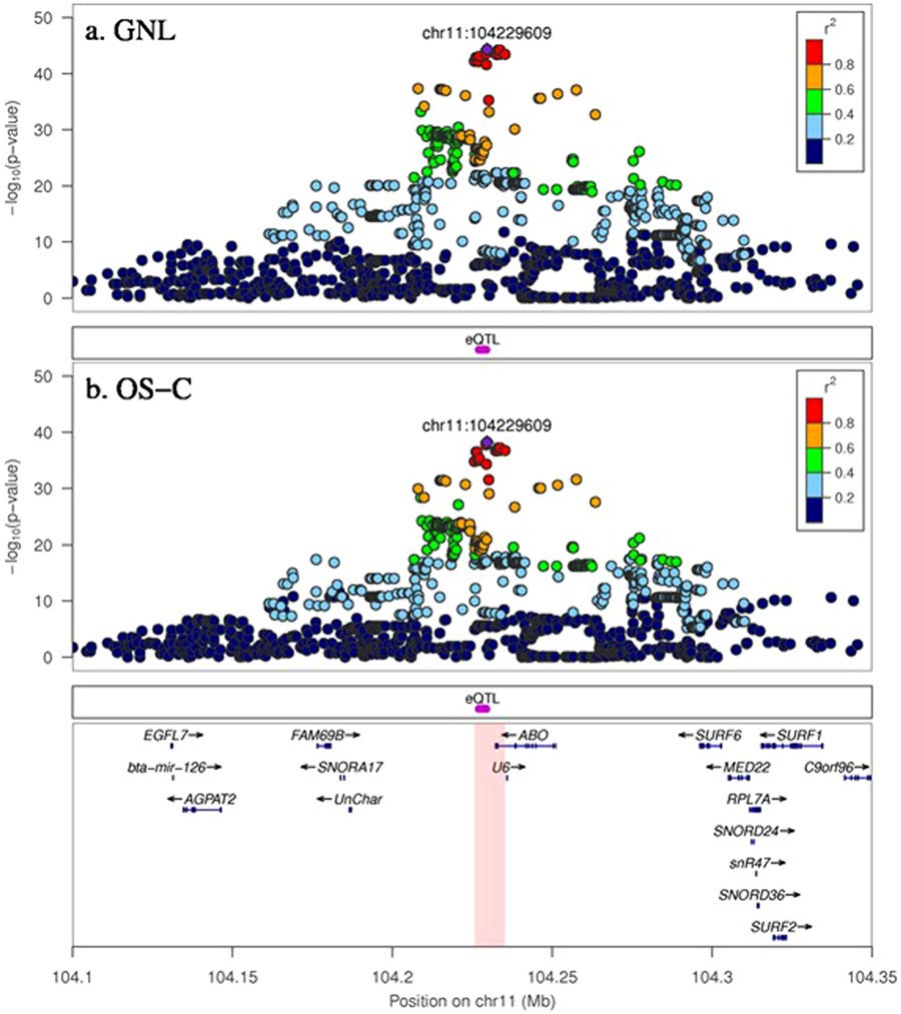
GWAS results with sequence variants showing a candidate gene region for GNL and OS-C. GNL and OS-C share a major QTL effect around the pink highlighted region which also overlaps with the most significant eQTL variants affecting ABO gene expression. In each plot the variant with the top −log_10_ (*p*-value) is shown by a purple diamond (with bp position label). The strength of LD (*r*^2^) between this top variant and all others is colour coded.

The fine-mapping results in Fig. 3 demonstrate that GNL and OS-C share the same major QTL effect on Chromosome 11. The most significant SNP (104, 229, 609 bp) for both traits is just 1908 bp downstream of the ABO gene which codes for an enzyme involved in the oligosaccharide biosynthesis. The −log_10_ (*p*-value) was 44 (GNL) and 38 (OS-C) for this top sequence variant, while in GWAS using HD SNP genotypes the most significant SNP had a lower −log_10_ (*p*-value) of 37 (GNL) and 31 (OS-C). The top sequence variant accounted for 78% and 84% of the genetic variance in GNL and OS-C respectively (Table 4), indicating that this or another variant in strong LD, is responsible for most of the genetic variation in both traits. The “eQTL” analysis of sequence variants associated with RNA transcript expression of the ABO gene, revealed a tight cluster of 14 sequence variants (between 104, 227, 111 and 104, 229, 385 bp) with the highest −log_10_ (*p*-value) of 7.06 for this gene (Fig. 3 and Table S-2, Supporting Information). This overlaps the region of the most significant SNP in Fig. 3, which strengthens the evidence that the underlying causal variant may be a regulatory variant in this intergenic region controlling ABO gene expression.

**Table 4:** Genomic information for the most significant GWAS sequence variants (multiple variants listed where they had equally significant *p*-values). Genes listed are those closest to all genic/intergenic SNP that were in linkage disequilibrium of *r*^2^ > 0.8 with the most significant SNP.

Closest genes in QTL region^a^ (Chromosome)	Most significant sequence variant position (bp)^b^	Sequence variant annotation	OS	Direction of minor allele effect	Genetic variation explained (%)
**ABO** (11)	104229609	Downstream (1908 bp from ABO)	GNL, OS-C	+	78% (GNL) 84% (OS-C)
**ST3GAL6** CPOX (1)	42593589 42593643 42596236	Intergenic Intergenic Intergenic	3′-SL	+	33%
RSPH10B CCZ1 PMS2 AIMP2 ANKRD61 EIF2AK1 (25)	38529260 38541187 38541547 38544511	Missense (RSPH10B) Intronic (RSPH10B) Synonymous (RSPH10B) Intronic (RSPH10B)	OS-A	−	12%
KAZN (16)	53653341 53653496 53653712 53653953 53654074 53654125 53654187 53654618 53654713 53654869	Intronic Intronic Synonymous Intronic Intronic Intronic Intronic Intronic Intronic Intronic	OS-B	+	11%
ANKRD31 (10)	6491671 (ANKRD31)	Intronic	OS-B	+	10%
ATRNL1 GFRA1 (26)	36764962	Intergenic	OS-B	+	9%

^a^Genes in bold are known to be involved in OS metabolic pathway.

^b^Multiple SNP listed for cases where more than one variant had equal *p*-values due to perfect LD between variants (*i*.*e*. *r*^2^ = 1). ABO: transferase A, alpha 1-3-*N*-acetylgalactosaminyltransferase; transferase B, alpha 1-3-galactosyltransferase. ANKRD61: ankyrin repeat domain 61. ANKRD31: ankyrin repeat domain 31. AIMP2: aminoacyl tRNA synthetase complex-interacting multifunctional protein 2. ATRNL1: attractin-like 1. CCZ1: vacuolar protein trafficking and biogenesis associated homolog. CPOX: coproporphyrinogen oxidase. EIF2AK1: eukaryotic translation initiation factor 2-alpha kinase 1. GFRA1: GDNF family receptor alpha 1. KAZN: kazrin, periplakin interacting protein. PMS2: postmeiotic segregation increased 2. RSPH10B (alias BT.24455): radial spoke head 10 homolog B. ST3GAL6: ST3 beta-galactoside alpha-2,3-sialyltransferase 6.

**Figure 4:**
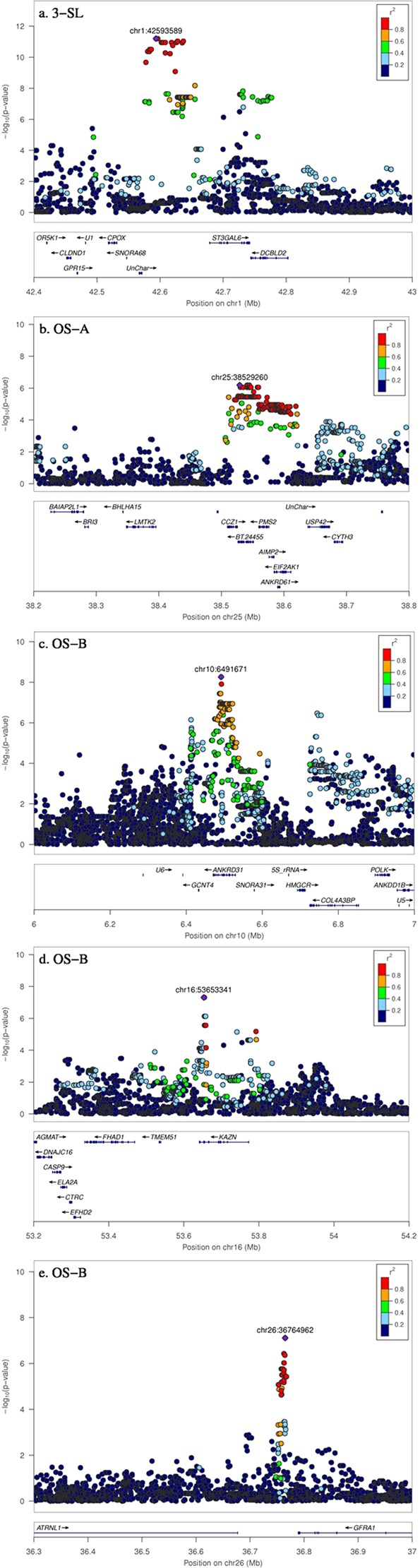
GWAS results with sequence variants showing fine-mapped QTL regions for 3′-SL (**a**) OS-A (**b**) and OS-B (**c**–**e**). The variant with the top −log_10_ (*p*-value) is shown by a purple diamond (with bp position label). The strength of LD (*r*^2^) between this top variant and all others is colour coded.

The most significant sequence variants for 3′-SL on Chromosome 1 (Fig. 4a) were upstream of genes ST3GAL6 and CPOX and also close to a small nucleolar RNA (SNORA68), suggesting that a causal variant in this intergenic region could be involved in regulating gene expression. Furthermore, the enzyme produced by the ST3GAL6 gene (β-galactoside α-2,3-sialyltransferase) is the key enzyme for production of 3′-SL and the most significant SNP is in strong LD with other SNP around this genic region (Fig. 4a). The most significantly associated variant explains 33% of the genetic variance, indicating a major effect on 3′-SL. However, no strong eQTL effects were detected for either ST3GAL6 or CPOX genes.

The most significant sequence variant for OS-A was on chromosome 25 (Fig. 4b) and explained 12% of the genetic variance (Table 4). For OS-A it is difficult to pinpoint a particular candidate gene: there are six genes in the chromosome region around the most significant variant and including those in very high LD (*r*^2^ > 0.8). Again, no significant eQTL effects were observed for these genes. For the OS-B trait there were three main QTL peaks on chromosome 10, 16 and 26 (Fig. 4c–e) and together they explain 30% of the genetic variance (Table 4). Although the most significant sequence variant is an intronic SNP in the ANKRD31 gene, it is also very close to the GCNT4 gene (Fig. 4c) that codes for the enzyme glucosaminyl-N-acetyl transferase 4, which is involved in milk oligosaccharide biosynthesis. There are no clear candidate genes for the OS-B trait on chromosomes 16 and 26 (Fig. 4d,e).

The presence of major QTL effects that explain a large proportion of genetic variance for several traits (GNL, OS-C, 3′-SL and OS-B), suggests that a simple strategy of marker-assisted selection (MAS) could be implemented to increase the abundance of these OS in bovine milk. Therefore, the estimated size of the major QTL effects was used to determine the potential for genetic improvement if animals were selectively bred to carry two copies of the favourable QTL alleles (Table 5). The QTL allele frequencies of the 332 experimental cows were found to be very similar to that of the Australian Holstein population generally (obtained from a large sample of industry animals available from DataGene Ltd, Melbourne, Australia). Obviously, the less common the favourable allele, the higher the potential for genetic improvement in these traits; noting that the minor allele generally showed the favourable effect.

**Table 5:** Predicted QTL effects and potential genetic improvement from marker-assisted selection (MAS) for traits GNL, OS-C, 3′-SL and OS-B.

Chromosome: Position	OS	MAF^a^ in this study (N = 332)	MAF in general Holstein population (N = 37678)	Effect size (arbitrary unit)	Current genetic average in population^b^	Potential genetic average from MAS^c^	Potential genetic improvement (fold) from MAS^d^
Chr11: 104229609	GNL	0.35	0.39	4724310	3684962	9448620	2.6
Chr11: 104229609	OS-C	0.35	0.39	247826	193304	495652	2.6
Chr1: 42593589	3′-SL	0.23	0.22	1062120	467333	2124240	4.5
Chr16: 53653341	OS-B	0.12	0.16	103766	33205	207532	6.3
Chr10: 6491671	OS-B	0.42	0.55	62639	68903	125278	1.8
Chr26: 36764962	OS-B	0.06	0.06	132046	15846	264092	16.7

^a^MAF: Minor Allele Frequency.

^b^Genetic average based on the marker effect and Hardy-Weinberg equilibrium genotype frequencies in the general Holstein population.

^c^Potential average based on selection for the entire herd carrying only the favourable alleles.

^d^Difference between the current genetic average due to the favourable mutation and the potential genetic average if all animals were selected to carry 2 copies of the favourable alleles.

## Discussion

Although over 40 OS have been identified in bovine milk, most of them are present at trace levels. Only the 12 most abundant species that can be reliably quantified without enrichment were surveyed in the first instance. These species are composed of 3–6 monomers with a molecular mass ranging from 500–1200 Da. In addition, half of them contain a sialic unit and thus are anionic. A strong correlation was observed between the abundance of some of the species, suggesting they are likely to share common steps in the biosynthesis pathway.

Cow to cow variation in the content of all major OS has been observed in our previous study^11^, and such variation was found to be temporally reproducible during the entire milking season for some OS species^24^. This prompted us to investigate the heritability and genetic architecture of OS accumulation in bovine milk.

We exploited sequence variants to fine-map six major candidate gene regions and putative causal mutations for five OS species. These OS included one high-abundance species (3′-SL), two intermediate-abundance species (GNL and OS-A) and two low-abundance species (OS-B and OS-C). It is worth noting that it is likely that more QTL of minor influence would be detected by increasing the size of the mapping population and/or by refining the phenotype data. Therefore, the list of QTLs found in this study is by no means exhaustive, but highlights some major gene effects.

The sequence GWAS fine-mapped a major QTL effect for GNL and OS-C which also overlapped a strong eQTL region that affected the expression of the ABO gene. The most significant SNP for OS-C and GNL was not among the most significant SNP in the eQTL region (14 variants were equal top because they were in perfect LD: Table S-2) but was within 224 to 2498 bp of the top eQTL SNPs. The RNAseq analysis was done on a subset of 107 cows, so it is possible that the LD between SNP and a causal mutation could change compared to in the 332 cows measured for OS. It is not possible to unequivocally determine the real causal mutation from this type of study because imputed sequence data inevitably has a low level of errors, meaning that the most significant variant is not always the causal variant^25^. Based on previous work we expect that the accuracy of imputation for the most significant sequence variants (Table 5) is approximately 0.9 where MAF ≥ 0.1^25^. Also, using only the association study results, it is not possible to distinguish the true causal variant from those in strong LD. However, our results suggest that the causal mutation may be a regulatory variant in this narrow intergenic region that controls the expression of the ABO gene.

The ABO gene, codes for α 1-3-N-acetylgalactosaminyltransferase and α 1-3-galactosyltransferase, the former being the key enzyme for the synthesis of GNL from lactose. OS-C contains one extra Gal unit as compared to GNL and this structural similarity implies that GNL is likely to be the precursor of OS-C. This may explain the co-localisation of QTLs detected for these two species. The most significant sequence SNP at 104, 229, 609 bp was also previously reported as being a putative causal mutation affecting overall milk protein yield in dairy cattle^26^. The allele that increased the GNL and OS-C abundance also increased milk protein yield (a desirable outcome). Additionally, the ABO gene was most highly over-expressed in lactating bovine mammary tissue when compared to 17 other bovine tissues^27^. The ABO gene determines the blood group of an individual in humans and blood groups were the first genetic markers in cattle as well. For example, the association between blood groups and the fat percentage of the milk in cattle was reported by Rendel^28^.

The most significant SNP for 3′-SL was fine-mapped close to a strong candidate gene (ST3GAL6) that codes for α 2-3-sialyltransferase: the key enzyme for the production of 3′-SL from lactose^29^. It is interesting to note that no QTL was identified for 6′-SL, an isomer of 3′-SL, but this may be due to lack of power because 6′-SL is at a lower abundance than 3′-SL. In the case of OS-A and OS-B, the functions of the candidate genes that encompass the most significant SNPs are not known to be directly related to OS synthesis except for GCNT4. GCNT4 codes for glucosaminyl (N-acetyl) transferase 4 and is one of the key enzymes involved in biosynthesis of milk OS. Interestingly, of all the genes close to the QTL for 3′-SL, OS-A and OS-B (Table 4), only ST3GAL6 and GCNT4 showed significant differential expression in lactating mammary tissue compared to 17 other bovine tissues.

For the remaining 7 major OS, no large QTLs were identified in this study. This is surprising given the structural similarity across all the major OS but is likely to reflect either a lack of power for the GWAS, given the number of sampled animals and/or lower accuracy in phenotyping of these traits. Nearly all OS are synthesised from lactose by successively adding various monomer units at different positions mediated by specific transferases^30^. The large difference in abundance across OS species of the same monomer number suggests a remarkable difference in the activity of various transferases involved in OS synthesis.

Although some simple OS could be produced *in vitro* with the use of appropriate transferases^31^, the possibilities of increasing the level of intrinsic OS in milk through herd management and/or genetic selection of cows have not been thoroughly explored. QTLs with moderate to large effects were detected for four of the OS species (GNL, OS-C, 3′-SL and OS-B), accounting for 30 to 84% of genetic variance. These are preliminary estimates that need to be confirmed in an independent population because the effect sizes may be overestimated due to the so called “Beavis effect”^32^ or “winner’s curse” common in GWAS. However, this indicates that a simple MAS strategy based on the described variants could more than double the OS abundance in milk (Table 5). We have also developed genomic predictions using all HD genome-wide markers in a single model (“genomic selection”: results not shown), but we need to phenotype more animals to adequately determine the accuracy of these whole-genome predictions. Although it is likely that the MAS approach will be equally accurate at this stage due to the presence of major QTL effects, once these alleles are fixed there may be more benefit in a genomic selection approach.

It is worth mentioning that the OS content is not known to be correlated with other components of milk (*e*.*g*. fat and protein) or animal health and performance traits (*e*.*g*. mastitis and fertility). There was no overlap of our OS QTL with those previously published for lactose^21^. This is expected because the relatively high abundance of lactose in milk relative to OS means that this important precursor should not become a rate limiting factor for OS synthesis. Although it seems unlikely that an increase in some OS species through MAS would have any negative impact on key dairy traits, this warrants further investigation. Equally, it would be important to better understand the genetic relationships between the concentration of different OS species. The OS species remain a minor component of milk even after a substantial increase of some, so the overall physical properties and flavour of milk is not expected to be altered.

In conclusion, this is the first study on heritability and genetic architecture of bovine milk OS abundance using sequence variants. A total of six genomic regions were fine-mapped on five chromosomes, affecting five of the 12 major OS. Among the major OS species detected, the accumulation of GNL and OS-C was found to be largely controlled by a single QTL; a dramatic increase in the content of these OS by marker assisted selection can thus be expected. QTLs accounting for 33% and 30% of variation were detected for 3′-SL and OS-B respectively, suggesting that genetic selection should also be effective in improving the concentration of these two OS in bovine milk.

## Materials and Methods

### Cows, herd management and milk sample collection

All experimental cows were maintained in the research Department of Economic Development, Jobs, Transport and Resources’ Ellinbank herd in Victoria, Australia. The experiment received animal ethics approval from the Agricultural Research and Extension Animal Ethics Committee of the Department of Economic Development, Jobs, Transports and Resources, Victoria, Australia. The experimentation was conducted in accordance with the Australian Code of Practice for the Care and Use of Animals for Scientific Purposes^33^. Cow diet varied through the milking season but most of the cows’ nutrient intake was derived from grazed pasture, supplemented with bought in feedstuff as required.

A total of 360 multiparous Holstein cows that calved in late winter/early spring were used in this study. The experiment was conducted over three years (2013, 2014 and 2015), with 120 cows participating each year. Milk samples were collected each year in three batches (40 animals per batch) over the period of mid-October to late-November. So, a total of 9 batches of samples (B1–B3 for year 2013, B4–B6 for year 2014 and B7–B9 for year 2015) were collected in this study. On each sampling occasion, the total milk from the afternoon and morning milking was collected into test buckets, pooled for each cow and a subsample taken for analysis. Milk samples were transported to the laboratory on ice and kept at −80 °C before analysis.

### Phenotyping

OS fraction was isolated from diluted raw milk using an ultra-filtration method and the filtrate used directly for LC-MS analysis. The detailed sample preparation procedure was as previously described^11^.

An Agilent 1290 UPLC system coupled to an LTQ-Orbitrap MS (Thermo Scientific) was used for OS quantification. Chromatographic separation of OS was achieved using a HILIC Kinetex column (150 × 4.6 mm, 2.6 µm, Phenomenex) maintained at 30 °C. The mobile phase was composed of 5 mM aqueous ammonium formate (A) and acetonitrile containing 0.1% formic acid (B). The flow rate was 0.8 mL/min and the elution started with 5% A for the first 3 min and then increased to 30% A from 3 to 17 min. The total run time was 26 min for each analysis. MS instrumental settings for OS analysis were as previously described^11^. All OS were detected in negative ion mode as their deprotonated ions. Due to the lack of standards for most OS, relative quantification was carried out for all the major OS. Peak area (after normalisation by the internal standard) was used as a measure for the relative abundance of each OS across all samples.

### Genotyping

The 360 cows were originally genotyped using either the Illumina BovineLD (~7,000 SNP array: https://sapac.illumina.com/products/by-type/microarray-kits/bovineld.html) or BovineSNP50 (~50,000 SNP array: http://www.illumina.com/products/by-type/microarray-kits.html) BeadChips. Those animals with low-density genotypes were then imputed by DataGene Ltd (Melbourne, Australia) as part of their routine genetic evaluations in Australia to the standard Illumina BovineSNP50 BeadChip using a reference population of more than 50,000 animals. The imputed BovineSNP50 genotypes comprised 39,756 SNP that passed quality control. These imputed and the real BovineSNP50 BeadChip genotypes were then imputed to the high density BovineHD BeadChip (800,000 SNP array). The reference population used for this imputation totalled 2155 animals with real genotypes for 632,003 SNP on the BovineHD BeadChip that passed a range of quality control filters following^34^.

After the initial GWAS, the genotypes of the same Holstein cows were imputed to whole genome sequence variants for each entire chromosome that showed strong associations with one or more of the OS traits. For imputation to sequence, we used a reference set of 645 sequenced dairy bulls from Run 5 of the 1000 Bull Genomes Project^35^. These included mainly Holstein breed (450) and several minor breeds including Jersey, Scandinavian Red and Guernsey. Sequence variants were only imputed if there were 4 or more copies of the minor allele observed among the reference bulls. The software used for each imputation step was FImpute using default parameters but not including pedigree^36^.

Finally, a principal component analysis (PCA) was conducted using the imputed BovineHD genotypes of the 360 animals (identified as Holstein breed) to check for outliers from the main genetic group based on the first two principal components. To improve the quality of the association studies, 28 outliers were removed so that 332 animals remained.

### Genome-wide association analysis (GWAS) and heritability estimates

The regression analysis model used in the GWAS tested the association of each SNP with each OS trait:

1 $${\bf{y}}={\bf{X}}{\boldsymbol{\beta }}+{\rm{Z}}{\bf{u}}+wa+{\bf{e}}$$

where **y** is the vector of phenotypic OS records of *q* individuals; **β** is the vector of fixed effects (sample batch number ranging from 1 to 9); **X** is a design matrix relating phenotypes to their fixed effects; **u** is the vector of animal effects where ${\bf{u}}\sim N(0,{\bf{G}}{{\rm{\sigma }}}_{g}^{2})$, **G** is the *q* × *q* genomic relationship matrix (based on HD genotypes) between pairs of individuals and ${{\rm{\sigma }}}_{g}^{2}$ is the additive genetic variance, and ***Z*** is the incidence matrix; *w* is the vector of animal genotypes at SNP_*i*_ coded as 0, 1 or 2 (representing the genotypes aa, Aa or AA) and *a* is the effect of the SNP; e is the vector of residual errors. The GWAS was conducted using GCTA software^37^. For this association analysis, HD SNP and sequence variants were only included if their minor allele frequency in the study cows was above 0.05 to avoid spurious false positives due to very rare alleles.

The heritability (h^2^) estimates were calculated in GCTA software using the same model as detailed above (Eq. 1) but without fitting the single SNP effect (“*a*”). That is, the genomic relationship was used to estimate the genetic variance (h^2^ was estimated as the ratio of this genetic variance to phenotypic variance).

Additionally, to assess the precision of GWAS, we report the false discovery rate (FDR) and quantile-quantile (Q-Q) plot based on the GWAS *p*-values. The Q-Q plot is a graphical representation that determines to whatdegree the observed GWAS *p*-values for each SNP deviate from the expected value (null hypothesis) with a theoretical χ^2^ distribution.

The FDR evaluates the rate of type I errors in null hypothesis testing: that is, evaluates the proportion of results at a given *p*-value threshold that are likely false discoveries. For the GWAS analysis, we calculated the FDR at four different *p*-value thresholds (*T*): *p* < 0.000001, *p* < 0.00001, *p* < 0.0001, and *p* < 0.001 by applying the following equation^38^:

$$FDR=\frac{T(1-s)}{s(1-T)},$$

where *T* is the threshold *p*-value from GWAS, *s* is the proportion of significant SNPs with *p*-values smaller than *T* relative to the total number of tests (*i*.*e*. *s* = number of significant SNPs divided by the total number of SNPs in the data).

### Gene expression QTL (eQTL) study

As part of a larger study^39^, we undertook an RNA sequence (RNAseq) analysis to quantify RNA transcript levels of candidate genes to determine if specific sequence variants were highly associated with transcript levels. Briefly, blood was sampled from a subset of 110 animals from those that were measured for OS. RNA was extracted from white blood cells and RNAseq libraries prepared and sequenced on a HiSeq™ 3000 (Illumina) in a 150- cycle paired end run. Sequence reads were trimmed and filtered of poor quality bases and sequence reads. Paired RNA reads for each sample were aligned to the Ensembl UMD3.1 bovine genome assembly using TopHat2^40^ allowing for two mismatches. Alignment files (.bam) for white blood cell libraries with >12.5 million read pairs (after quality control filtering) also having >80% mapping rate were retained for gene count matrix generation. Gene counts were produced using the python package HTSeq^41^ and were combined to form a gene by sample count matrix. This count matrix was then normalised to take into account library size using the R software package, DESeq^42^. After quality control, RNAseq data for 107 cows was included in the eQTL study. Only genes that were expressed in more than 25 cows were included for further analysis to avoid spurious associations due to very low read counts. There were 11,178 genes remaining in the analysis.

A GWAS was then undertaken where the normalised counts of RNA transcripts for candidate genes were the ‘phenotypes’ (*y* in eq. 1). As in eq. 1, each sequence variant was tested for association with the gene expression level (“eQTL”), testing only variants on the same chromosome as the gene under test.

## Supplementary information

Supplementary

## Data Availability

The DNA sequences (1000 Bull genomes Project) are available at NCBI BioProject: PRJNA238491 and PRJNA431934. The RNAseq data (White Blood Cells) is available under NCBI BioProject: PRJNA305942.
