# Supplementary material for: Fine-mapping sequence mutations with a major effect on oligosaccharide content in bovine milk
Source: Sci Rep. 2019 Feb 14;9:2137. doi: 10.1038/s41598-019-38488-9 (PMC6376028; doi:10.1038/s41598-019-38488-9)
Supplement: Supplementary file 1 — Supplementary [file 41598_2019_38488_MOESM1_ESM.pdf]

Supplementary Information

Fine-mapping sequence mutations with a major effect on oligosaccharide content in bovine milk

Zhiqian Liu[1,a], Tingting Wang[1,a], Jennie E. Pryce[1,2], Iona M. MacLeod[1], Ben J. Hayes[1,3], Amanda J. Chamberlain[1], Christy Vander Jagt[1], Coralie M. Reich[1], Brett A. Mason[1], Simone Rochfort[1,2*] and Benjamin G. Cocks[1,2]

Table S-1. Pairwise correlation (r) in abundance across the major OS species (n = 360).

	Triose	3'-SL	6'-SL	GNL	6'-SLN	DSL	OS-A	OS-B	OS-C	OS-D	OS-E
Triose											
3'-SL	0.15										
6'-SL	0.22	0.42									
GNL	-0.06	-0.26	0.00								
6'-SLN	0.34	0.19	**0.63**	0.16							
DSL	0.11	0.18	0.31	0.22	**0.69**						
OS-A	-0.03	0.15	0.20	0.15	0.38	0.57					
OS-B	-0.32	0.13	0.15	0.03	0.19	0.26	**0.65**				
OS-C	0.12	-0.10	0.05	**0.84**	0.31	0.34	0.15	0.02			
OS-D	-0.46	0.01	0.05	-0.15	0.06	0.32	0.44	**0.95**	-0.09		
OS-E	0.05	0.14	0.15	0.13	0.52	0.57	0.33	0.15	0.20	0.04	
OS-F	0.33	0.06	0.20	0.26	**0.63**	**0.78**	0.44	0.03	0.39	0.02	0.53

3’-SL: 3’-sialyllactose

6’-SL: 6’-sialyllactose

6′-SLN: 6′-sialyl-N-acetyllactosamine

DSL: disialyllactose

GNL: *N*-acetylgalactosaminyllactose

OS-A: 3’-sialylgalactosyllactose

OS-B: lacto-*N*-pentaose

OS-C: lacto-*N*-neotetraose

OD-D: di-*N*-acetylhexosaminyltriose

OS-E: 3′-glycolylneuraminyllactose

OS-F: 3'-sialyl-*N*-acetylglucosaminyllactose

Table S-2. RNAseq eQTL analysis of ABO gene expression, testing all sequence variants on chromosome 11. The position of the most significant SNP associated with the ABO RNA transcript abundance is listed with the -log *p*-value.

Chromosome:position (bp)	-log *p*-value
Chr11:104225654	6.42
Chr11:104226169	6.42
Chr11:104226184	6.42
Chr11:104226396	6.42
**Chr11:104227111**	**7.06**
**Chr11:104228091**	**7.06**
**Chr11:104228120**	**7.06**
**Chr11:104228217**	**7.06**
**Chr11:104228291**	**7.06**
Chr11:104228607	6.71
**Chr11:104228721**	**7.06**
**Chr11:104228726**	**7.06**
**Chr11:104228735**	**7.06**
**Chr11:104228842**	**7.06**
**Chr11:104228949**	**7.06**
**Chr11:104228983**	**7.06**
**Chr11:104229223**	**7.06**
**Chr11:104229261**	**7.06**
**Chr11:104229385**	**7.06**
Chr11:104232298	6.42
Chr11:104232312	6.42
Chr11:104232319	6.42
Chr11:104232354	6.42
Chr11:104232725	6.19
Chr11:104232763	6.19
Chr11:104235463	6.42
Chr11:104235480	6.42
Chr11:104237486	6.42
Chr11:104237602	6.42
Chr11:104237645	6.42
Chr11:104237825	6.42
Chr11:104237889	6.42
Chr11:104238052	6.42
Chr11:104238257	6.42
Chr11:104238465	6.42
Chr11:104238576	6.42
Chr11:104251713	6.04
